# From Innovation to Conservation: Sustainable Pathways in Vitreoretinal Practice

**DOI:** 10.1155/joph/5598625

**Published:** 2025-12-04

**Authors:** Lorenzo Iuliano, Marco Gonfiantini, Francesco Bandello, Paolo Lanzetta

**Affiliations:** ^1^Department of Medicine-Ophthalmology, University of Udine, Udine, Italy; ^2^Department of Ophthalmology, IRCCS San Raffaele Scientific Institute, Vita-Salute University, Milan, Italy; ^3^Istituto Europeo di Microchirurgia Oculare - IEMO, Udine, Italy

**Keywords:** carbon footprint, disposable, fluorinated gases, global warming potential, greenhouse gas, sustainability, vitreoretinal surgery

## Abstract

Vitreoretinal surgery, while clinically advanced and increasingly prevalent, presents a substantial environmental burden due to the widespread use of disposable instruments, fluorinated gases, and energy-intensive infrastructure. This narrative review explores sustainability in vitreoretinal practice, identifying key contributors to its carbon footprint and outlining strategies for mitigation. The environmental impact of tamponade agents—particularly sulfur hexafluoride (SF_6_), with its extremely high global warming potential—is emphasized through data from recent multicenter studies. Additional contributors include inefficient gas delivery systems, single-use surgical instruments, and the disposal of persistent toxic substances. The article also addresses how expanding surgical indications and reliance on single-use technologies have compounded ecological costs. Evidence-based strategies for improving sustainability are discussed, including the use of air tamponade in selected cases, gas dilution protocols, adoption of single-use canisters, reusable instrumentation, optimized OR workflows, and teleophthalmology pathways that reduce unnecessary postoperative visits. Sustainable vitreoretinal surgery is presented not only as a possibility but as a clinical, ethical, and environmental imperative, achievable without compromising patient outcomes.

## 1. Introduction

Sustainability is essential to ensure a balanced coexistence between healthcare progress and environmental stewardship. Healthcare systems are responsible for approximately 4%-5% of total global CO_2_-equivalent (CO_2_e) emissions, ranking among the top industrial sectors worldwide [1–3]. Within hospitals, operating rooms (ORs) alone account for about 13% of total energy consumption and 30%–70% of hospital waste [4]. In ophthalmology, the environmental burden is amplified by procedure volume, with over 25 million cataract surgeries and several million vitreoretinal procedures performed annually [1]. As healthcare systems strive to reduce emissions, ophthalmology emerges as a uniquely impactful specialty due to its reliance on high-volume procedures and advanced technologies. The same factors that underpin its success (precision instruments, disposable materials, and complex supply chains) also make it a significant contributor to healthcare-related emissions.

Many components of the carbon footprint are shared across all ophthalmic surgeries. These include emissions from travel (involving patients, staff, and materials), electricity consumption (for the OR, heating/ventilation/air conditioning [HVAC] systems, and water), production and manufacturing (of instrumentation, linens, and disposable supplies), chemical manufacturing, and waste management services. These domains have been especially well-characterized in the context of cataract surgery, which due to its global prevalence, has often served as the benchmark for environmental assessments in ophthalmology [4]. Reported per-case carbon costs vary widely between high- and low-income settings, ranging from approximately 182 kg CO_2_e in a UK tertiary center to only 6 kg CO_2_e in an Indian facility [5, 6]. This contrast underscores the influence of healthcare infrastructure, regulation, and supply chain efficiency on sustainability metrics.

Cataract surgery has been the prototype for ophthalmic sustainability analyses, but vitreoretinal surgery presents additional challenges. It is inherently more resource-intensive, relying on high-energy systems, advanced visualization technologies, and many single-use devices. Moreover, vitreoretinal procedures frequently use fluorinated gas tamponades such as sulfur hexafluoride (SF_6_), hexafluoroethane (C_2_F_6_), and octafluoropropane (C_3_F_8_), which are among the most potent greenhouse gases, with global warming potentials (GWPs) up to 23,900 times that of CO_2_. As the global population ages and indications for surgery expand, these cumulative impacts demand scrutiny [7].

Despite growing awareness of environmental sustainability in cataract and general surgery, the literature specifically addressing vitreoretinal surgery remains limited. Only a few multicenter or regional studies have quantified its environmental burden, highlighting wide variability in carbon emissions depending on surgical technique and gas delivery systems. This paucity of evidence underscores the need for dedicated analyses and structured recommendations for more sustainable vitreoretinal practice [5, 7, 8].

Identifying sustainable alternatives that preserve patient safety and clinical efficacy is imperative for the future of ophthalmic care. In this article, we explore the critical intersection between innovation, surgical practice, and environmental responsibility within the realm of vitreoretinal surgery.

## 2. Methods

This review was conducted as a narrative synthesis of the available literature on sustainability in vitreoretinal surgery. A structured search was performed in PubMed, Scopus, and Web of Science databases up to May 2025, using combinations of the keywords (“vitreoretinal surgery,” “sustainability,” “carbon footprint,” “environmental impact,” “greenhouse,” and “ophthalmology”). Priority was given to peer-reviewed original research articles, systematic reviews, multicenter studies, and authoritative guidelines. Reference lists of included articles were manually screened to identify additional relevant studies. Given the scope and heterogeneity of the available evidence, a formal risk of bias assessment was not performed.

We also aimed to ensure geographic diversity by including evidence from low- and middle-income countries (LMICs) and non-European regions. In addition to primary studies, we incorporated recent area-wide narrative reviews that synthesize sustainability data across ophthalmic subspecialties and settings, thereby supporting a global perspective on best practices and implementation barriers.

To structure our discussion, we address six key questions that frame the current and future challenges of sustainability in this field:

1. Why sustainability matters in vitreoretinal surgery?
2. How the environmental impact can be quantified?
3. How vitreoretinal surgery affects the environment?
4. Which innovations have increased environmental burden?
5. How to mitigate the environmental impact?
6. What defines sustainable vitreoretinal surgery?

### 2.1. Why Sustainability Matters in Vitreoretinal Surgery?

Vitreoretinal surgery, while often shorter in duration than other surgical procedures, carries a substantial environmental footprint due to its high frequency, technical complexity, and reliance on high-impact materials.

Globally, an estimated 3–5 million vitreoretinal procedures are performed each year, generating on average 30–60 kg CO_2_e per case, primarily from fluorinated gases and single-use instruments. This corresponds to a cumulative 150,000–300000 tons CO_2_e annually, comparable to the yearly energy consumption of several thousand households [1, 5, 6].

As the global population ages and access to eye care expands, the volume of vitreoretinal surgeries continues to rise. Recent registry data show that rhegmatogenous retinal detachment (RRD) and macular hole (MH) procedures have increased by approximately 25%–40% over the past decade, driven by demographic ageing, the increasing prevalence of diabetes and myopia, and expanded surgical accessibility in developing regions [2, 6, 9]. These trends reinforce the urgency of addressing sustainability in vitreoretinal care.

Fluorinated gas tamponades represent one of the most impactful contributors. Their 100-year GWPs (GWP_100_) reach 23,900 for SF_6_, 12,200 for C_2_F_6_, and 8830 for C_3_F_8_, as reported by multicenter analyses [5, 6, 8]. A single RRD repair using SF_6_ emits around 60 kg CO_2_e, while MH repair with C_3_F_8_ averages 32 kg CO_2_e [6]. Moreover, each procedure produces 2–4 kg of single-use plastic waste, mostly from trocar sets, tubing, and drapes [4].

• High environmental burden: Although often short in duration, each vitreoretinal procedure involves intensive resource use. Each vitreoretinal case emits 30–60 kg CO_2_e, equivalent to driving a car for 250–300 km [5, 6]. Cumulatively, this results in a significant carbon footprint across healthcare systems.
• Global reach: Vitreoretinal procedures are performed worldwide with increasing frequency due to population aging and better access to specialized care. With 3–5 million cases annually, the cumulative footprint approaches 150–300 kt CO_2_e [1].
• High-volume impact: Even minor improvements in sustainability per procedure can result in large cumulative benefits given the high global surgical volume. A 10-kg reduction per procedure could prevent 30,000–50000 tons CO_2_e per year [4].

Compared with cataract surgery, vitreoretinal procedures are less frequent but significantly more resource-intensive, due to longer operating time, high-energy endoillumination, and reliance on high-GWP tamponades [4–6]. These characteristics justify a dedicated focus on sustainability within vitreoretinal practice.

### 2.2. How the Environmental Impact Can Be Quantified?

Quantifying the impact of surgical practices is essential for tracking improvements and establishing benchmarks. Key approaches include carbon footprint analysis, GWP, material waste audits, and life-cycle assessment, which together provide a comprehensive measure of environmental impact. These frameworks have been applied in ophthalmology and vitreoretinal surgery to compare technologies, assess sustainability interventions, and identify high-impact domains [1, 4, 6, 8].

• GWP: This standardized index quantifies how much heat a greenhouse gas traps in the atmosphere relative to CO_2_. Over a 100-year horizon (GWP_100_), fluorinated gases commonly used as intraocular tamponades (SF_6_, C_2_F_6_, and C_3_F_8_) have values of 23,900, 12,200, and 8830, respectively, reflecting their high persistence and climate effect [5, 7].
• Carbon footprint (CO_2_e): This metric estimates total greenhouse gas emissions from a given process or product. With regards to surgery, it encompasses gas use, instrument manufacturing, energy consumption, and waste management.

For vitreoretinal surgery, the carbon footprint is typically calculated using the formula: CO_2_e (kg)=mass of gas(kg) × GWP_100_.

For example, 1 kg of SF_6_ with a GWP_100_ of 23,900 results in 23,900 kg CO_2_e. In vitreoretinal surgery, reported emissions range between 30 and 60 kg CO_2_e per procedure, depending on gas type, delivery system, and local infrastructure [6, 8]. By comparison, cataract surgery ranges from 6 kg CO_2_e in low-resource settings to 182 kg CO_2_e in tertiary hospitals [4, 5], illustrating how surgical context influences sustainability metrics.

• Material waste audits: Quantitative assessments involve collecting and weighing the total solid waste per surgery. In vitreoretinal surgery, it ranges typically 2–4 kg, predominantly composed of plastic packaging, tubing, gloves, syringes, and drapes [4]. These analyses guide targeted interventions, such as replacing single-use trays with reusable sets or adopting recyclable alternatives.
• Life Cycle Assessment (LCA): A more comprehensive method, LCA, evaluates environmental impacts across the entire lifespan of a product—from raw material extraction and manufacturing to use and disposal. LCA enables comparisons between reusable and disposable devices, highlighting that reusables can reduce per-case emissions by 40%–70%, contingent on sterilization efficiency and energy mix [1, 5]. Applying LCA to vitreoretinal practice thus supports evidence-based decision-making toward a more sustainable, low-emission model of care.

These tools allow ophthalmic departments to monitor trends, evaluate the benefits of sustainability initiatives, and guide procurement decisions toward greener alternatives.

### 2.3. How Vitreoretinal Surgery Affects the Environment?

Vitreoretinal surgery affects the environment through multiple interconnected pathways, including the production and use of high-GWP fluorinated gases, the energy demands of OR and machines, and the generation of single-use waste. Understanding these sources of impact provides the foundation for targeted interventions to reduce emissions and promote sustainability across ophthalmic practice. This section reviews each of these contributors, emphasizing their quantitative relevance and modifiable nature.

#### 2.3.1. Fluorinated Gases

Fluorinated gases are essential tamponade agents used to maintain retinal apposition during postoperative healing. Their physical properties (low solubility and high surface tension) make them indispensable in complex retinal detachment and macular surgery. However, these same characteristics lead to extreme atmospheric persistence, making them a major source of greenhouse emissions in vitreoretinal care.

Sulfur hexafluoride (SF_6_), hexafluoroethane (C_2_F_6_), and octafluoropropane (C_3_F_8_) are the standard intraocular tamponades. These gases are among the most potent greenhouse gases, with GWPs that far exceed CO_2_. Their usage in eye surgery, while representing a small portion of overall emissions, has a disproportionate environmental impact due to their longevity and potency [7, 8].

Sulfur hexafluoride (SF_6_) has a GWP_100_ approximately 23,900 times greater than that of CO_2_. In practical terms, the release of a single 30-mL canister of SF_6_ corresponds to the CO_2_ emissions of driving a typical car for over 200 km [5, 6, 8, 10]. Given the widespread use of these gases, their cumulative contribution to the ophthalmic carbon footprint is substantial.

A landmark multicenter study by Moussa et al. has provided some of the most comprehensive data to date on the carbon footprint of common vitreoretinal procedures involving fluorinated gases [6]. Across 4877 surgeries performed at three UK tertiary centers over 4 years, the study quantified a total emission of 284.2 tons of CO_2_e mass—equivalent to the annual consumption of over 30,000 L of gasoline.

The most environmentally impactful indications were RRD and MH repairs, accounting together for over 75% of total emissions. On average, RRD surgeries produced 60 kg and MH repairs 32 kg CO_2_e mass. SF_6_ was the dominant contributor: Although used in less than 40% of cases, it accounted for nearly 70% of the total CO_2_e mass. This is due to its extremely high GWP (GWP100 = 23,500) and long atmospheric lifetime. In contrast, C_2_F_6_ and C_3_F_8_ had significantly lower per-case emissions, the latter being the less impactful.

Utilization patterns also differ by region; for example, a center in India reported C_3_F_8_ use in ∼70% of vitreoretinal cases. Published per-procedure estimates align with our figures, ranging ≈32 kg CO_2_ for MH repair to ≈60 kg CO_2_ for RRD [11].

#### 2.3.2. Disposable Tools

Modern vitrectomy relies heavily on disposable instruments, such as trocar cannulas, vitrectomy probes, and light fibers. While these enhance sterility and efficiency, they also generate large volumes of medical waste [5].

#### 2.3.3. Inefficient Gas Delivery

Traditional large gas cylinders often result in partial usage with substantial waste. Transitioning to smaller single-use canisters can significantly reduce emissions, although they also have embedded carbon costs from production [6, 8].

#### 2.3.4. Toxic Liquids

Perfluorocarbon liquids, while essential in complex retinal surgeries, are chemically persistent and pose challenges in disposal and environmental safety [5].

While fluorinated gases remain the dominant contributor, other elements also add meaningfully to the environmental impact.

ORs account for approximately 13% of total hospital energy use and 30%–70% of hospital waste, largely due to illumination systems and HVAC units required to maintain positive-pressure sterile environments [1, 4]. Energy use during vitrectomy itself averages 0.5–1.2 kWh per procedure, depending on microscope and console specifications [8].

Waste generation also represents a tangible component of the footprint. As mentioned above, each vitreoretinal procedure produces approximately 2–4 kg of solid waste, that account for 60%–80% of the total waste mass [4].

Although less impactful in CO_2_e terms than gas emissions, these materials remain a modifiable source of environmental burden and a practical entry point for sustainability interventions through re-engineering, reuse, and recycling initiatives.

In summary, the environmental impact of vitreoretinal surgery is driven by the combined effects of fluorinated gas emissions, energy-intensive operating environments, and high volumes of single-use waste. Addressing each of these domains is essential for transitioning toward a lower-carbon model of ophthalmic care.

### 2.4. Which Innovations Have Increased Environmental Burden?

Over the past 2 decades, technological and procedural advances have profoundly improved the safety, precision, and outcomes of vitreoretinal surgery.

The introduction of high-speed vitrectomy machines and integrated fluidics platforms has optimized cutting precision and intraoperative stability, but these systems are energy-intensive and rely on proprietary single-use cassettes. Similarly, disposable light probes, trocar and cannula sets, and chandelier systems have improved visualization and surgical ergonomics, yet generate substantial quantities of nonrecyclable waste. Advances in digital visualization platforms and intraoperative OCT have enhanced surgical accuracy but increased power consumption, particularly in centers performing multiple procedures daily.

These developments have indeed increased the specialty's environmental burden by raising energy demands, expanding single-use material consumption, and intensifying reliance on high-GWP gases. Understanding how progress translates into ecological cost is essential for balancing clinical excellence with environmental stewardship [4, 5, 8, 9].

Advances in surgical technique have also broadened indications for pars plana vitrectomy to include novel therapies such as gene therapy for inherited retinal dystrophies or treatment of hemorrhagic age-related macular degeneration. These clinical expansions, alongside improved access to retinal care and growing procedural safety, have markedly increased case volumes worldwide. This rise, although beneficial to patients, proportionally amplifies energy use, gas consumption, and medical waste, thereby expanding the environmental footprint of vitreoretinal practice [5, 9, 12].

As highlighted in recent sustainability reviews, technological progress in ophthalmology follows a paradoxical pattern: Each innovation enhances surgical safety and efficacy but often at the cost of greater resource consumption. Recognizing this trade-off is the first step toward designing innovations that advance both clinical outcomes and environmental responsibility.

### 2.5. How to Mitigate the Environmental Impact?

Reducing the environmental footprint of vitreoretinal surgery requires a multifaceted approach that combines evidence-based surgical choices with system-level optimization.

Several key strategies, ranging from the adoption of lower-impact surgical techniques and tamponade agents to the reintroduction of reusable instruments and streamlined operating workflows, can substantially reduce emissions while maintaining surgical safety and efficacy [4, 6, 9].

#### 2.5.1. Surgery Choice

Scleral buckling and pneumatic retinopexy represent lower-impact alternatives for selected retinal detachments. Both avoid fluorinated gas use, disposable vitrectomy cassettes, and high-energy platforms, resulting in per-case emissions below 10 kg CO_2_e, compared to 30–60 kg CO_2_e for pars plana vitrectomy [5, 6, 10]. While not universally applicable, they remain valuable in younger patients and localized detachments.

#### 2.5.2. Tamponade Choice

Among vitreoretinal procedures, the choice of tamponade is a critical determinant of carbon footprint. Despite its advantages, air tamponade remains underused due to concerns about inferior or complex breaks. In carefully selected cases, particularly MH repair and superior retinal breaks, air tamponade is a viable and environmentally friendly alternative to fluorinated gases [13].

A 2023 meta-analysis of 2687 cases found no significant difference in primary reattachment rates between air and fluorinated gases (95.6% vs. 96.1%), even in inferior detachments [14].

A multicenter study in United Kingdom evaluating 3239 RRD cases found that air tamponade use at one center in 30% of cases led to a dramatic reduction in carbon footprint [12]. Specifically, mean CO_2_ emissions per patient were 1.9 kg, compared to 7.9 kg and 115.9 kg at centers using multiuse canisters and gas cylinders, respectively. When adjusted for a uniform 30 mL canister delivery system, air still achieved a 41.1%–47.0% reduction in CO_2_ emissions compared to fluorinated gases. Extrapolated to a national level, these findings suggest that using air for just 30% of RRD repairs could prevent up to 716.5 tons of CO_2_ emissions annually in the United Kingdom, equivalent to the electricity consumption of over 120 homes.

Despite these advantages, the adoption of air tamponade remains limited in many settings, primarily due to concerns regarding its adequacy for tamponading inferior retinal breaks or more complex detachments. These concerns are understandable; however, growing evidence from recent meta-analyses supports the efficacy and safety of air even in a broader range of indications. Taken together, these findings position air tamponade as one of the most impactful and evidence-based sustainability interventions in vitreoretinal surgery, meriting broader implementation in appropriately selected cases.

#### 2.5.3. Gas Dilution Strategies

Lower concentrations of C_2_F_6_ and C_3_F_8_ can provide adequate tamponade while significantly reducing the associated GWP. At our center, we have routinely used a halved concentration of C_3_F_8_ (6%) since 2010, with no appreciable compromise in clinical outcomes. This approach has proven effective in achieving satisfactory tamponade behavior, particularly in cases of retinal detachment. Supporting this strategy, Lin Teh et al. demonstrated that a 6% concentration of C_3_F_8_ achieves approximately 70% vitreous cavity fill at postoperative Day 7, comparable to traditional concentrations [10]. These findings support the broader adoption of gas dilution protocols as a practical, low-impact measure to enhance the environmental sustainability of vitreoretinal surgery.

#### 2.5.4. Gas Delivery

One of the most significant and often overlooked contributors to the carbon footprint of vitreoretinal surgery is the method by which fluorinated gases are delivered. A major multicenter study in United Kingdom by Moussa et al. compared the environmental impact of three gas delivery systems: traditional large gas cylinders, 75 mL canisters, and 30 mL single-use canisters [7]. The findings were striking: gas cylinders produced 40 times more CO_2_e emissions per patient than 30 mL canisters, with a mean emission of 124.8 kg CO_2_ per patient when cylinders were used, compared to just 3.17 kg with 30 mL canisters. This dramatic difference was primarily due to significant underutilization and wastage linked to the fixed volume and expiry dates of large cylinders.

These findings underscore the crucial role that procurement decisions and theater logistics play in reducing the ecological impact of surgical care. Encouragingly, single-use canisters are increasingly available in Europe and offer a viable, environmentally friendlier alternative for many surgical settings.

#### 2.5.5. Reusable Instruments

Reprocessing instruments (where safety regulations allow) can significantly reduce waste. The reintroduction of reusable surgical instruments has indeed shown promising results: forceps, scissors, and vitrectomy handles can be safely sterilized for multiple cycles, reducing per-case emissions by 40%–70% [1, 4]. Updated sterilization protocols, especially in centers powered by renewable electricity, enhance sustainability without compromising infection control.

Evidence from high-volume LMIC programs indicates that carefully validated reuse pathways can be both safe and impactful on waste and emissions. The Aravind Eye Care System reports endophthalmitis rates ≤ 0.02% in > 300 k cataract cases despite extensive reuse under standardized protocols, and a Thai series of 12,989 vitrectomies with recycled devices reported an endophthalmitis incidence of 0.10%, comparable to published ranges. While policy constraints in high-income countries often limit reuse, these data demonstrate that risk-controlled reprocessing can meaningfully reduce environmental burden without compromising outcomes [9, 11].

#### 2.5.6. Green OR Practices

Implementing environmentally conscious OR protocols can significantly reduce waste and resource consumption.

A key component of green OR initiatives is the introduction of effective recycling programs. Despite common misconceptions, many items used during ophthalmic surgery are recyclable if properly segregated. These include glass and plastic bottles (such as irrigating solution containers), plastic drapes, instrument pouches, paper packaging, and cardboard boxes. Establishing clear waste streams and staff education programs ensures that recyclable materials are appropriately diverted from general waste. Some institutions have also partnered with vendors to reclaim and recycle surgical instrument trays and devices. Collectively, these practices not only reduce environmental impact but also help to instill a culture of sustainability within surgical teams.

Reducing the vitreoretinal surgical tray is another practical measure. Studies indicate that up to one-third of items opened for PPV remain unused, representing unnecessary material and energy expenditure [4]. Regular audits of tray composition, combined with surgeon-driven customization, can yield immediate waste reductions.

Finally, hospital-level interventions such as optimizing customized packs, minimizing nonessential draping (e.g., the microscope), and adopting energy-efficient HVAC and lighting systems have been shown to lower OR energy use by 15%–25%, with measurable CO_2_e savings [1, 4]. Education of surgical teams and integration of sustainability metrics into quality improvement programs are essential to ensure durable impact.

#### 2.5.7. Telematic and Decentralized Care

The integration of teleophthalmology and decentralized care pathways offers a powerful avenue for reducing the environmental impact of vitreoretinal surgery. Recent discussions in the literature have questioned the universal necessity of routine postoperative Day 1 visits, particularly following uneventful pars plana vitrectomy procedures. Studies have shown that in low-risk patients—those without significant intraoperative complications, undergoing macular surgery or floaterectomy, and with good preoperative visual potential—the yield of postoperative Day 1 visits is minimal in terms of identifying adverse events. In these cases, delayed in-person follow-up or virtual check-ins may be both safe and effective [15, 16].

Implementing teleophthalmology tools such as video consultations, patient-reported symptom checklists, and remote visual acuity apps allows clinicians to triage patients effectively and reduce unnecessary travel and clinic visits.

This approach not only enhances patient convenience and reduces carbon emissions associated with transportation but also streamlines clinic operations, allowing resources to be concentrated where they are most needed. Ultimately, a risk-stratified, personalized approach to postoperative care can maintain patient safety while contributing meaningfully to sustainability goals.

### 2.6. What Defines Sustainable Vitreoretinal Surgery?

Sustainable vitreoretinal surgery integrates clinical excellence with environmental responsibility, aiming to minimize emissions and waste while maintaining high standards of safety and efficacy. It is grounded in three complementary dimensions: evidence-based clinical practice, resource efficiency, and organizational sustainability. It is not defined by a single action but by a set of interrelated practices that collectively reduce the environmental burden of ophthalmic care.

Central to this approach is the thoughtful selection of intraocular tamponade agents. Whenever clinically appropriate, air or low-GWP gases should be preferred over high-impact alternatives (such as SF_6_). This alone can markedly reduce carbon emissions associated with surgery.

In addition, the transition from single-use instruments to validated reusable tools offers another opportunity to curtail waste. Although regulatory and sterility concerns must be addressed, studies suggest that in selected contexts, reusability can significantly lower the solid waste footprint and associated costs.

The physical setting of the surgery also matters. Practical measures include optimizing OR workflows, minimizing nonessential draping, and rationalizing material use. Recent audits have quantified the benefits of these interventions: optimizing OR case sequencing and HVAC downtime can reduce per-procedure energy consumption by 15%–25%, while rationalizing draping and disposable coverage can decrease solid waste by 30%–40% without compromising sterility [1, 4].

The EyeSustain network and multicenter analyses in ophthalmology further demonstrated that workflow redesign and tray optimization yield combined carbon savings of approximately 0.5–1.0 kg CO_2_e per case and tangible cost reductions [9, 17].

Beyond the OR, sustainability also involves how care is delivered to patients. Streamlined patient pathways, including telemedicine for follow-ups and diagnostic evaluations, reduce travel-associated emissions and logistical inefficiencies [1, 17].

Programs in LMICs illustrate that streamlined workflows and decentralized/tele-enabled care can reduce patient travel and per-case emissions while maintaining outcomes, offering transferable principles for HIC settings [11].

Ultimately, sustainable vitreoretinal surgery is a dynamic balance: maintaining or enhancing patient outcomes while minimizing the environmental cost of delivering that care. It requires commitment at both the individual surgeon level and institutional policy level, integrating evidence-based strategies into daily practice and long-term planning.

## 3. Conclusion

Sustainability in vitreoretinal surgery is no longer an aspirational ideal: It is an ethical imperative. The ophthalmic community must embrace environmentally responsible choices, from gas selection to instrument reusability and OR design. By doing so, we not only preserve our planet but also ensure that future generations inherit a healthcare system that is as sustainable as it is effective.

## Data Availability

Data sharing is not applicable to this article as no datasets were generated or analyzed during the current study.
